# The Impact of Evidence-Based Dialogic Training of Special Education Teachers on the Creation of More Inclusive and Interactive Learning Environments

**DOI:** 10.3389/fpsyg.2021.641426

**Published:** 2021-03-03

**Authors:** Alfonso Rodríguez-Oramas, Pilar Alvarez, Mimar Ramis-Salas, Laura Ruiz-Eugenio

**Affiliations:** ^1^Department of Sociology, University of Barcelona, Barcelona, Spain; ^2^Department of Education, Research Methods and Evaluation, Universidad Pontificia de Comillas, Madrid, Spain; ^3^Department of Theory and History of Education, University of Barcelona, Barcelona, Spain

**Keywords:** evidence-based dialogic teacher education, special educational needs, Mexico, interactive learning environments, inclusion

## Abstract

In the international context of a progress toward more inclusive educational systems and practices, the role of Special Education teachers is being transformed. From an inclusive perspective, these professionals increasingly support students and their teachers in the mainstream classroom, avoiding segregation. However, Special Education teachers often struggle to reach and support all students with special needs and their teachers to provide quality inclusive education. For this reason, more research is still needed on in-service training strategies for the inclusion of students with special needs that effectively translate into evidence-based school practices that improve the education of all students. This article analyses the impact of two evidence-based dialogic training programs of Special Education teachers working in mainstream schools carried out in Mexico during the 2018–2019 school year. Through in-depth interviews with participants, it was identified how, after the training, teachers increasingly grounded their actions on scientific evidence and promoted interactive learning environments that improved the educational inclusion of their students with special needs. This training also became the venue to make evidence-based educational actions available to other students without special needs, improving the quality of education provided to all students.

## Introduction

In the current social scenario, it is increasingly important to promote a high-quality education as a key requirement to prepare all students—including those students with diverse needs—for the acquisition of the basic skills that are necessary to actively participate in society. The Sustainable Development Goal 4 (SDG4) included in the United Nations 2030 Agenda for Sustainable Development (United Nations, 2015) highlights the need to “ensure inclusive and equitable quality education and promote lifelong learning opportunities for all.” Inclusive education involves “transforming education systems so they can better respond to learners' diversity and needs (...) fulfilling the right to education with equality (...) not only to access, but also to participation and achievement of all students” (United Nations, 2016, p. 44). Thus, despite the existing differences in the definition of what inclusion means across countries (Cooc, 2019), inclusive education is today recognized as the appropriate educational strategy to promote the education of students with Special Educational Needs (SEN) or disabilities in the international scenario (Malinen et al., 2013; Chao et al., 2017; De Haro et al., 2019).

In this context of increasing support to the inclusion of children with Special Educational Needs into mainstream education (European Commission, 2019), the need to going beyond the integration of students with diverse needs in the general classroom should be noted. This becomes the necessary condition for guaranteeing a truly inclusive educational response that makes possible an adequate participation and learning for all students. In this way, as it is warned in the Global Education Monitoring report 2020 (UNESCO, 2020), including students with Special Educational needs in mainstream schools that are not prepared to provide them with an adequate inclusive response can end up leading to a worsening of the situations of exclusion experienced by these students. As recent studies point out, the mere integration of students with diverse needs into the general classroom does not immediately translate into the creation of better opportunities for interaction and collaborative work that are fruitful for the whole class (Pinto et al., 2019).

In relation to the pathways to improve the educational response for all students, prior research has pointed toward the creation of interactive learning environments as an effective strategy to transform schools into more inclusive spaces, in which shared opportunities for learning and social participation between students with special needs and their peers can emerge (Garrote et al., 2017). Different studies reveal that maximizing opportunities for contact and social interaction between students with special needs and their peers can help alleviate the obstacles to participation and social acceptance that students with diverse needs often suffer (Avramidis et al., 2018), while it can also increase their opportunities for academic development (Pinto et al., 2019). In a similar vein, recent research has provided evidence of the social impact obtained by the implementation of Successful Educational Actions (Duque et al., 2020) aimed at increasing the learning and social interactions among students with diverse needs, though the participation of family and community members in various learning activities. Interestingly, Duque et al. (2020) explored venues to develop more interactive learning environments both when including students with special needs and their typically developing peers in general classrooms, as well as among those students with special needs enrolled in Special Education schools.

Moving toward a more inclusive education through the promotion of interactive learning environments often implies reexamining and expanding the role of Special Education teachers. From the focus on responding individually to the needs of students with special needs—which often implies withdrawing them from the general classroom, thus limiting their opportunities for social interaction with their typically developing peers—recent conceptions of the role of specialist teachers are evolving toward more inclusive approaches, aimed at promoting opportunities for collaborative work and social participation among diverse peers within the general classroom (Rose and Shevlin, 2020). Consequently, if the goal is to transform classrooms into interactive environments that offer the maximum opportunities for learning and development for all students, it is necessary to better prepare and support teachers to offer adequate scaffolding to students, so that high-quality interactions among diverse students can take place (Hong et al., 2020). In fact, the pedagogical competence of professionals working in the field of Special Education has been highlighted as a determining factor in promoting interaction between peers to improve the communicative and social skills of children (Syrjämäki et al., 2017).

Despite the evidence supporting the creation of interactive learning environments that allow students with special needs to increase their opportunities for learning and socialization within the general classroom (Vetoniemi and Kärnä, 2019), the educational practice with these students in segregated classroom settings is still a persistent trend in many countries (Somma, 2020). This reveals the need to better align the educational practices carried out in schools with the evidence-based knowledge about the most effective ways to promote a more inclusive response to the learning and developmental needs of all students (Mitchell and Sutherland, 2020). Some recent studies (Brock et al., 2020) have warned that, despite the existence of evidence-based knowledge in the field of Special Education, there is a significant gap between the available research-based knowledge and the practices implemented in schools (Cook and Odom, 2013).

Improving teacher education and professional development can be a decisive factor to address this gap. The scientific literature has long pointed to the importance of teacher education and professional development, and its impact on improving the quality of education (Darling-Hammond et al., 2017). Regarding the role of teacher education in enhancing inclusion (Florian and Camedda, 2020; Ní Bhroin and King, 2020), the Salamanca Statement (UNESCO, 1994) highlighted the recruitment and training of educational personnel as one of the key axes to advance toward a more inclusive approach to Special Educational needs. Among other contributions, the Declaration stressed that one of main challenges for achieving inclusion was to provide in-service training to all teachers, considering the varied and often difficult conditions in which they provide services. Likewise, it was pointed out that training for in-service teachers should be developed, when possible, at the school level, through interaction with the trainers and with the support of distance education and other self-instruction techniques.

When it comes to examining the challenges to improve the professional development needs of Special Education teachers, Cooc (2019) identified two international trends: many schools around the world face a shortage of teachers with competency in the field of Special Education, and a significant proportion of teachers express the need for more professional development, especially those who work with a bigger share of students with special needs. If we look at the characteristics that should be present in teacher education and professional development initiatives in the field of Special Education, some studies have highlighted the need to pay attention to the self-efficacy of teachers when it comes to providing an educational response to students with Special Educational Needs or disabilities (Sharma et al., 2012; Malinen et al., 2013). Not in vain, increasing teachers' sense of efficacy is related to the use of the best educational practices, as well as with the improvement of attitudes toward diversity and inclusion (Sharma et al., 2012). Furthermore, the scientific literature has pointed to the power of collaborative work between different types of teachers (Malinen et al., 2013; Robinson, 2017), as well as among teachers and other key actors (families, other professionals) when developing plans to improve the educational response to students with Special Educational Needs (Ní Bhroin and King, 2020). Furthermore, it should be noted that, when it comes to support teachers to getting evidence into use in the field of education (Gorard et al., 2020; Joram et al., 2020), this must go beyond sharing research trends among the teaching staff and encouraging teachers to make more use of research outcomes. It also implies promoting changes in the “research culture” at the district/regional level, so that teachers can develop a stronger sense of “agency” to take part of decision-making regarding the educational agenda in their schools or districts (Joram et al., 2020).

The present study aimed to contribute to the scholarship on how to support teachers working in the field of Special Education to get evidence-based knowledge into use in their school practice, in order to improve the learning and development opportunities of all their students. More specifically, our study analyzed the impact of two dialogic teacher education initiatives developed in Mexico City (Mexico), aimed at preparing in-service teachers working in the field of Special Education to implement evidence-based educational actions to promote more interactive learning environments for all students, including students with Special Educational Needs enrolled in general classrooms.

## Materials and Methods

### Research Questions

The present study aimed to address two research questions:

- What has been the impact of the participation in two evidence-based dialogic teacher education programs for a group of in-service teachers in Mexico as regards the transformation of their educational response to students with Special Educational Needs enrolled in mainstream schools?
- What are the main strategies that have allowed participants to translate this evidence-based knowledge to their educational practice, with the goal of transforming their classrooms into more interactive learning environments for all students?

### Context of the Study

#### Implementation of Successful Educational Actions: The Relevance of Dialogic Teacher Education

The study focused on analyzing the impact of two teacher education programs developed in Mexico City (Mexico). These training actions were put into practice within the framework of implementation of a broader educational program, entitled Schools as Learning Communities (Garcia-Carrion, 2016). This project, first developed in Spain, consists in the transformation of schools into Learning Communities, through the implementation of a set of so-called Successful Educational Actions (Flecha, 2015). Successful Educational Actions are evidence-based actions aimed at promoting dialogue and interaction among students, together with the participation of family and community members in learning activities and decision-making at school. Because of the impact obtained by the Schools as Learning Communities in Spain, which was analyzed by the FP6 INCLUD-ED research project (2006–2011) (Flecha, 2015), in recent years, the Successful Educational Actions have been transferred to a wealth of schools in different countries (Rodriguez Mello and Marini Braga, 2018; Soler et al., 2019; Diez-Palomar et al., 2020).

Among the Successful Educational Actions analyzed by the INCLUD-ED project, it is worth highlighting two of them which are expressly aimed at transforming the classroom into an interactive learning environment for all students: Interactive Groups and Dialogic Literary Gatherings. In Interactive Groups, the classroom is split into small heterogeneous groups of students (in terms of language, learning level, ethnic origin, etc.). Each small group works collaboratively on different classroom activities, accompanied by an adult volunteer, who is responsible for stimulating interaction between all students as a means to help them complete the different tasks. During a classroom session, each group completes as many activities as groups are formed in the classroom. Through this type of classroom organization, all students are encouraged to participate in learning activities on an equal footing with the rest of the class, thanks to the mutual help among peers and the support of adult volunteers (Valero et al., 2018; Zubiri-Esnaola et al., 2020). In turn, Dialogic Gatherings consist of transforming the classroom into an interactive learning environment in which students read and share their views on world literature books, in an environment marked by egalitarian dialogue and respect for the diversity of opinions. The Dialogic Gatherings support the development of communication skills and school-relevant knowledge, while creating opportunities for students to build shared meanings about socially relevant issues (Lopez de Aguileta et al., 2020). As stressed by Aguilera-Jiménez and Prados-Gallardo (2020) the implementation of both Successful Educational Actions allow teachers to intensify the interactions among all students, not only in terms of quantity (maximizing the opportunities for cooperation among students), but also in terms of quality and diversity (promoting solidarity and mutual help among students with diverse needs, with the support of adult volunteers).

One decisive feature for the rigorous transferability of the Successful Educational Actions to new schools and contexts is the dialogic teacher education (Roca et al., 2015), which seeks to promote evidence-based dialogic training among teachers, allowing them to better sustain their educational practice on the most relevant educational theories and the latest scientific developments in the field. Dialogic teacher education promotes a first-hand approach to primary scientific sources among teachers, in a context marked by egalitarian dialogue between the participants, aimed to promote reflection on how to better translate evidence-based knowledge into their teaching practice. Prior research (Roca et al., 2015; Rodriguez et al., 2020) examined the impact of dialogic teacher education in Spain, showing that the participation in these training initiatives allowed teachers to build shared knowledge on how to provide a more effective response to the problems found in their school practice.

#### Dialogic Teacher Education for Special Education Teachers in Mexico

Regarding the current scenario of inclusive education in Mexico, in recent years, different efforts has been undertaken to ensure quality education for all (Hrusa et al., 2020), aimed at transforming educational practices and policies in the field of Special Education in Mexico to promote an inclusive education (Garcia-Cedillo et al., 2014). However, research shows that a greater drive is needed to translate the inclusion discourse present in the latest reforms to the educational practices put into practice in schools (Garcia-Cedillo, 2018). Among the pending challenges to move toward the successful implementation of inclusive education in Mexico, the need for greater collaboration among all stakeholders in the education of students with Special Educational needs—teachers, administrators, families and the community as a whole—has been pointed out. Likewise, the need to advance in the implementation of educational practices and programs aimed at providing equitable and high-quality education for all students, with and without disabilities, has been highlighted (Garcia-Cedillo et al., 2014). Furthermore, improving teacher professional development has also been identified as a critical step to foster equity and inclusion (Hrusa et al., 2020).

Against this backdrop, the present study explored how dialogic teacher education can equip teachers with evidence-based knowledge, to give a new impetus to inclusive practices in schools, in order to transform their classrooms and schools into more interactive learning environments. More specifically, we analyzed the impact of two evidence-based dialogic teacher education programs for in-service teachers in Mexico City launched in 2018, during the end of the 2017–2018 and the beginning of the 2018–2019 school years. These two programs were delivered by a team of educational professionals trained in the scientific bases of the Schools as Learning Communities project, who work for the civil organization Vía Educación and the Natura Institute in Mexico. Since 2015, this team collaborates with local authorities to transfer the Successful Educational Actions to schools in the city. With this aim, in the last years they have organized numerous evidence-based dialogic training courses targeted at teachers and other education staff. Specifically, our study focused on the experiences of a group of teachers who work in the field of Special Education in Mexico City, which participated in one or both programs described below:

- **Initial dialogic professional development program:** Throughout 2018, a number of intensive training actions were carried out aimed at teachers, principals, school supervisors and technical-pedagogical advisors at various educational levels (from early childhood to secondary education), in order to train them on the scientific bases that underpin the Successful Educational Actions carried out in the Schools as Learning Communities project. Different evidence-based dialogic training initiatives were implemented, which included a 40 h online training program (which had an estimated participation of over 120 people in total, and a duration of 10 weeks), a 20 h in-person intensive training program for regular and Special Education staff (which had ~125 participants, and lasted one week), as well as a 25 h in-person training program specifically for Special Education professionals (which had ~200 participants and was carried out in three moments over 12 weeks; namely, a first moment, with a duration of 16 h, during the first week, a second moment, with a duration of 5 h, in the middle and, finally, a third moment, with a duration of 4 h in the last week of the training program). Despite having different formats and lengths, all the three initiatives fully covered the modules that comprise the “Raising Awareness” training course that teachers must receive prior to transform their schools into Learning Communities (Garcia-Carrion et al., 2017). Thus, the topics of the intensive trainings included the theory of Dialogic Learning and the bases of the Successful Educational Actions (Flecha, 2015), with a focus on the creation of interactive learning environments to promote the educational inclusion of all students, including students with Special Education Needs or disabilities.
- **Ongoing dialogic professional development program (dialogic pedagogical gatherings):** At the beginning of the 2018-2019 school year, a permanent teacher training seminar was created in Mexico City, based on the experience of the “On the Shoulders of Giants” seminars created in Valencia, Spain (Rodriguez et al., 2020). These are monthly encounters in which teachers and other educational professionals participate in dialogic pedagogical gatherings (Roca et al., 2015), with the aim of deepening on the theoretical foundations of the Successful Educational Actions and improving the educational practice in their schools. For that purpose, the participants read and debate, based on an egalitarian dialogue, the most important theoretical contributions of authors like Paulo Freire, Lev Vygotsky and Jerome Bruner, among others, as well as recent scientific articles published in high-profile journals and top research reports on education. Generally, these monthly encounters are divided into two parts: a first part, which is devoted to the discussion of the selected readings, and a second part, in which different committees are formed, which allow an in-depth discussion on different topics related to classroom practice which are of interest for the participants. The permanent seminar in Mexico City, which was open for anyone who completed any of the initial dialogic teacher education actions described above, had the participation of an average of 60 professionals, including teachers, principals, school supervisors and technical-pedagogical advisors, some of them working in the field of Special Education. The permanent seminar was active during the entire 2018-2019 school year, and sessions were held monthly, with a duration of 4 h per session.

### Data Collection

The study reported data collected through in-depth interviews with five teachers working with students with Special Educational Needs or disabilities enrolled in different mainstream schools in Mexico City (Mexico). The criteria used for the selection of participants were the following: (1) participants must be teachers in the field of special education, (2) which had attended at least one of the actions within the Initial dialogic professional development program and/or had been part of the permanent seminar (Ongoing dialogic professional development program), and (3) which had expressed the improvement of their students from the work carried out in dialogic training and the implementation of Successful Educational Actions. Table 1 describes the group of participants in the study, paying attention to their professional position, as well as their experience participating in dialogic teacher education. In order to preserve confidentiality and anonymity, all the names that appear in the study are pseudonyms.

**Table 1:** Participants in the study.

**Name**	**Age**	**Years in service**	**Professional task**	**Experience in dialogic teacher education**
Anita	42	20	School supervisor in the field of Special Education. She is the coordinator of a team in charge of the supervision of Special Education teachers working in a total of 42 early childhood, elementary and secondary schools.	Completed the 25 h in-person training program for Special Education professionals and then joined the permanent seminar, which she attended regularly throughout the year.
Nora	47	24	Technical-pedagogical advisor in the field of Special Education. She advises 15 kindergarten schools. She works under Anita's supervision.	Completed the 40 h online training program and then joined the permanent seminar, which she attended regularly throughout the year.
Diana	38	15	Technical-pedagogical advisor in the field of Special Education. She advises 8 kindergarten schools.	Completed the 25 h in-person training program for Special Education professionals and the 20 h in-person training program.
Miguel	53	26	Special Education teacher in a kindergarten school. He is advised by Diana.	Completed the 20 h in-person training program.
Roberta	44	21	Technical-pedagogical advisor in the field of Special Education. She serves 21 kindergarten schools.	Completed the 25 h in-person training program for Special Education professionals, the 40 h online training program and the 20 h in-person training program.

Data collection was performed in two stages between 2019 and 2020. Firstly, between July and August 2019, we conducted two paired in-depth interviews (one with Anita and Nora, and another one with Diana and Miguel), as well as one in-depth interview with Roberta. After the preliminary analysis of the information, it was considered suitable to delve into the views and perceptions of two of the research participants, which had been previously paired-interviewed, due to their significant involvement in the dialogic teacher education initiatives conducted, and because they could provide us information of special value (Read, 2018) to shed light on the impact of the training actions conducted. Therefore, a second round of fieldwork was planned and carried out in July 2020, which included two additional individual in-depth interviews, one with Anita and one with Nora. This allowed us to obtain a deep insight of the training experiences carried out, as well as on how taking part in dialogic teacher education contributed to transform the participants' educational practice toward their students with Special Educational Needs.

In order to ensure that the study followed the international ethical guidelines for conducting research with human beings, all participants were informed about the objectives and the characteristics of the research, as well as about their rights as participants, including the possibility of withdrawing from the study at any time. Furthermore, all participants in the study provided their informed consent to participate in the research. The study was fully approved by the Ethics Board of the Community of Researchers on Excellence for All (CREA).

### Data Analysis

In line with the two research questions posed, the data analysis was aimed at examining the impact of their participation in the dialogic teacher education programs on the teachers involved in the project. The audio recordings from the interviews were transcribed verbatim and analyzed, in order to allow a thorough exploration of participants' experiences, perspectives and views. From this preliminary analysis, a series of themes emerged that illustrate, firstly, the impact that participation in the dialogic teacher education programs had on the participants, in relation to their adoption of evidence-based knowledge, as well as to their vision of their role as teachers in the field of Special Education. Secondly, our analysis brought out the different ways in which participants have managed to translate the evidence-based knowledge gained through the participation in dialogic teacher education into their teaching practice, in order to transform their classrooms into more inclusive learning environments for all their students, including those with Special Education needs. Table 2 summarizes the main themes that emerged from the qualitative data analysis:

**Table 2:** Coding scheme.

**Category**	**Themes**
1. Impact of evidence-based dialogic teacher education on participants	1.1 Embracing evidence-based knowledge
	1.2 Rethinking the role of teachers in Special Education
2. Strategies to translating the evidence-based knowledge gained through dialogic teacher education into practice, to develop more interactive learning environments	2.1 Successful Educational Actions to promote more interactive learning environments for all
	2.2 Promoting the participation of the entire community
	2.3 Making dialogic training sustainable to keep improving practice

## Results

### Impact of the Participation in Evidence-Based Dialogic Teacher Education

First, the findings about the impact that the dialogic teacher education had on the teachers participating in our study are presented. The transformation of their conception of the role of specialist teachers, as well as their commitment to adopt evidence-based knowledge are discussed.

#### Embracing Evidence-based Knowledge

Dialogic teacher training was aimed at making teachers aware of the scientific bases that underlie the Successful Educational Actions (SEA), to promote their rigorous implementation in the field of Special Education. Notably, this evidence-based knowledge is aligned with the need to promote the inclusion of all students, as well as with the key role of interaction as a tool to promote learning the importance of interaction for learning and social participation of the Students with Special Needs in such inclusive settings. For so doing, an intensive initial training was established, aimed at introducing the theories and evidence that support the Successful Educational Actions to participants. The evidence-based dialogic approach that underpins this training program, which involves presenting primary scientific sources to teachers, meant for many teachers examining their prior educational practice in the light of the scientific evidence. Some participants exposed the initial difficulties to carry out this reflective analysis about their ways of teaching their students, because of their lack of experience in evidence-based training:

For me the project was rich, but at the same time it struck me because I said, I mean, what I was trained on, is it not supported? Have I been doing it wrong all these years? (Roberta, Technical pedagogical advisor, Special Education)
Of course, it was a shock because it is something you do not know. I have been a teacher for 25 years. And this was new to me. Totally different, it broke all the schemes (Miguel, Special Education teacher, Kindergarten).

In fact, the emphasis on putting evidence-based knowledge at the service of teachers, in the eyes of Roberta, contrasted with previous teacher professional development experiences, which used to be very focused on presenting “trendy” educational theories, without delving into their theoretical and practical foundations. In her view, that kind of teacher education, which usually run the risk of being replaced by other new approaches when political changes take place in educational administrations, do not have a profound impact on teacher practice:

This is not common. We find a lot about the latest methodology, a lot of popular methodologies, which are “in fashion,” you know? And that, depending on the six-year term, on the political moment in which you find yourself, you know that it will change. So, I feel that much of what we find as teachers is a bit of confusion, so to speak, because we know that we are going to acquire (knowledge on) what they give us, only for a short time. As soon as they change any person in a position, something else will come. So, they don't allow you to adhere to it or to embrace it, they never tell you about its foundations (Roberta, Technical pedagogical advisor, Special Education).

In contrast, the dialogic teacher education is based on presenting the results of the implementation of Successful Educational Actions, which are evidence-based actions that have been previously implemented in a sustainable way in many schools in very diverse contexts and countries. During the initial dialogic teacher training, all this accumulated knowledge was shared and discussed with teachers. In Anita's opinion, this allowed participants to obtain clear guidelines to start rethinking their practice, to transform it:

It is not something that you have to invent, the methodology is very clear, (...) it is actually rather that you respect that methodology when implementing it, so that then it achieves the results that have been already proven (Anita, Special Education supervisor).

A key aspect for the consolidation of the evidence-based approach beyond the initial dialogic teacher training was the development of dialogic pedagogical gatherings, which are spaces for horizontal continuous training, in which teachers meet to continue deepening their training. Through the reading and discussion of scientific sources, participants build new knowledge to keep on improving their practice. The fact of promoting these spaces for continuous training have been decisive for participants to make sense of evidence-based knowledge and embrace it:

This part of having read the books, of listening… because you put your experience, your experiences and so on at stake there, but when you listen to those of others it is like saying: I had not seen it from that perspective. And it has happened, for example, within the services, with the teachers, when we already talked about the readings, and at that moment there were some teachers who said: “I had never thought about it that way, and maybe I'll do it that way.” (Anita, Special Education supervisor).
But do you know what worked? The Dialogic Pedagogical Gathering. That was what made Linda and Laura (two teachers) convince themselves to put it into practice, to say, “Ok, I didn't want to at first, but if you give me the opportunity, and you come with me [to the classroom], I will.” (Diana, Technical-pedagogical advisor, Special Education).

#### Rethinking the Role of Teachers in Special Education

One of the fields in which dialogic teacher training has had the most decisive impact is the transformation of the participants' vision of their role as Special Education teachers. As described above, dialogic teacher education follows an inclusive educational approach, which aims to help teachers develop more interactive learning environments for all students through the implementation of Successful Education Actions. This approach contrasts with the more widespread model in Special Education, focused on providing an individual and differentiated response to students with Special Educational Needs or disabilities. This shift in perspective represented an important change for Special Education teachers and raised initial concerns among participants about the feasibility of implementing the project in classrooms serving diverse students. In the words of Roberta:

I fell in love with the project, (...) but at the same time, there were questions like “how are we going to connect it here in Special Education? How are (we going to manage) the difficult situations (...)? How are we going to let all the parents enter?” Those were questions that were being generated… (Roberta, Technical pedagogical advisor, Special Education).

In addition, the possibility of transforming classrooms into inclusive contexts aroused among the Special Education teachers the fear that their educational task with students would be blurred or could even disappear:

The point is that the changes that have occurred in the Special Education model have been complex for some teachers, because they have gone from working directly with the child, to now no longer be able to do so. So, for some it has been like taking away, to a certain extent, the tool they had to work with children (Nora, Pedagogical Advisor, Special Education).
Then suddenly I got into conflict and I told them: these 14 years that I have been a teacher have been of no use. Because at the end of the day in Special Education they have always told you... at the beginning of the school year we based (our work) on the characteristics and abilities of the children, then, from that diagnostic evaluation your work (is developed) throughout the school year. And when you told me that this was not supported (by evidence), I said: “I have not done anything right!” (Diana, Technical-pedagogical advisor, Special Education).

Overcoming these initial resistances involved creating opportunities for dialogue and meaning-making among participants, so that specialist teachers could see opportunities to redefine their role in supporting students' needs in an interactive learning environment. In this sense, the dialogic teacher training thus opened the door for teachers in the field of Special Education to rethink their vision about their own professional task, in line with the goal of transforming the classroom to maximize the opportunities for learning and social interaction of their students with Special Educational needs or disabilities in collaboration with their peers:

Sometimes I do believe that in Special Education we segregate (the students), we do not include (them). Being immersed in a school and realizing that we only serve this type of student, when we should be serving the entire school, is what limits us. But I do firmly believe that this type of educational action (...) opens the door to all of us (Diana, Technical-pedagogical advisor, Special Education).
Just the fact that you look at these other possibilities that allow you to work to favor that context, and that it is really going to have an impact on that student, and that you can really see it, that is like changing to another perspective: that you can do what you should, not focusing on the student with SEN, but that you really must see the environment, the community (Nora, Pedagogical Advisor, Special Education).

### Translating Evidence-based Knowledge into Practice

In what follows, we present the findings regarding the strategies employed by participants to translate the evidence-based knowledge acquired through their participation in dialogic teacher education to their teaching practice, in order to transform their classrooms into more inclusive interactive learning environments. Participant's efforts to implement the Successful Educational Actions, as a way to foster the learning opportunities of all their students, including those students with Special Educational Needs, together with the importance of promoting the family and community participation, as well as the need to guarantee the sustainability of the dialogic teacher education are illustrated.

#### Implementing Successful Actions to Promote More Interactive Learning Environments for All

When analyzing how the dialogic teacher training helped participants to start promoting changes in the schools in which they work, they emphasized the implementation of Interactive Groups and Dialogic Gatherings in their schools as the driving force for the transformation of their educational practice, aimed at building more interactive environments for all students, including those with SEN or disability. Promoting the implementation of these evidence-based actions meant, in the eyes of the participants, putting into practice a truly inclusive approach, thus favoring the participation of students with SEN in the learning activities:

When we were starting the school year, there was a lot of talk about inclusive policies, and all that stuff. But it was lip service, because really the teachers, in doing so, failed. But when Interactive Groups began to be held with children, very important changes occurred (Miguel, Special Education teacher, Kindergarten).
So (with), Interactive Groups, Dialogic Literary Gatherings, which is what has been implemented in this school year with the students, you can work on it with all the children. All students, regardless of their condition. Whatever the student, you see that they learn, that they participate and that the community is involved (Anita, Special Education supervisor).

To illustrate the changes in the classroom learning environment that took place from the implementation of Interactive Groups and Dialogic Literary Gatherings, Diana and Miguel brought up the case of Marcelo, a 5-year-old pupil with an intellectual disability and a family's history of abuse, enrolled in the 3rd year of Early Childhood Education:

This case attracted me in a special way because no one could control the poor kid. It has a very sad story (...) because the child, if we caught his attention, ran and got under the desk, as if to protect himself. Or he would run and crawl under his own chair. (...) (Miguel, Special Education teacher, Kindergarten).

Up to that point, the educational response to Marcelo's special needs had been focused on trying to control his behavior when in class, to the detriment of his learning objectives:

The teacher, she was already a senior, and she had a hard time recognizing Marcelo's strengths. She was more determined in ensuring that the child was sitting than in his learning. Or to have him coloring (during class), so that he would not disturb others (Diana, Technical-pedagogical advisor, Special Education).

In his second year at school, and after the participation of part of the school's teaching staff in dialogic teacher education, Interactive Groups began to be implemented in Marcelo's classroom. At that point, Miguel, as the Special Education teacher, proposed that Marcelo participate in the groups with the rest of his classmates. The participation in Interactive Groups gave Marcelo the opportunity to increase his social interactions with his peers aimed at the acquisition of learning objectives. In Miguel's eyes, the opportunity of taking part of the learning activities in an environment marked by mutual help and collaborative learning with their peers and an adult volunteer contributed to boost Marcelo's learning:

I said to the teacher: you know what? We are going to work on this with him. I didn't know... And we started working at Interactive Groups. The child already recognizes quantities and numbers from 1 to 10, he recognizes them, as soon as you ask him, he says them in a skipped way. And that happened because of the Interactive Groups that we did in mathematics (Miguel, Special Education teacher, Kindergarten).

Interestingly, they also highlighted how the participation in Interactive Groups meant a personal transformation for Marcelo: from being “very labeled (...) the one who hits (his classmates), the one who cannot stay still” (in Diana's words), he started to feel just like another member of the class, able to contribute to the classroom work, and to get help from his peers when needed. Hence, the implementation of Successful Educational Actions made it possible to transform the classroom climate in favor of a more stimulating environment for interaction and learning for all. Not only students with special educational needs benefited from this change, but the entire group:

With the parents who worked with the children (as volunteers) (…) the child went unnoticed. You wouldn't say “this kid has an intellectual disability.” Because he participated like the other children. (...) that was very shocking for me. And if the children themselves saw that he couldn't, they helped him.

It should be noted here that the implementation of evidence-based actions (Interactive Groups and Dialogic Literary Gatherings) meant an opportunity for participants to move from discourse to action when it comes to transforming their classroom practice to promote the full participation of students with Special Educational needs. This process required teachers to examine their own beliefs and expectations toward these students. Anita illustrates this change in perspective through the case of Marco, a 1st grade school student with a developmental disability, which caused him a speech delay, among other communication disorders. Anita explained how the student's evolution from his participation in Successful Education Actions led her and the rest of the teachers to realize their initial low expectations toward his learning possibilities:

We saw him, and I tell you we saw him because I (saw him that way) too, and I had to “eat my words,” because we saw him very far away, and then we said, “this little boy (it is enough), if he goes to school and socialize, and maybe he could learn to interact with his classmates…” Unfortunately, sometimes you resign yourself… (...) but no! When the (standardized) test was reapplied, (...) the child had already accessed literacy, in mathematical thinking the child had acquired the contents of the grade... and it was something that really surprised us a lot, because I must say that we didn't even realize when the student actually started to read! (....) With this student, our expectations, unfortunately, and yes, I accuse myself, because it wouldn't have to be that way, our expectations were very low to him.

#### Involvement of the Entire Community

Another key action carried out by participants to translate the evidence-based knowledge gained through the dialogic teacher training into their classroom practice with students with special needs was promoting the participation of family and community members in the school. Among the opportunities for the engagement of family members in the learning activities of students with SEN, the participants highlight the possibility that parents enter the classroom to cooperate as volunteers in the implementation of Interactive Groups. This allowed family members to get to know the educational situation of the student and their needs, while facilitating their communication with teachers:

Regarding other strategies that we carry out in Special Education, I think it has been a plus that, while you are applying the actions, the Interactive Groups or the Dialogic Literary Gathering, the parent is integrated. So, it doesn't require you to have an interview with the parent separately; the parent him/herself is realizing the needs (of the student), and you are not the spokesperson for what he/she should do with the child outside of (school), but it arises from the desire of the parent him/herself (Anita, Special Education supervisor).

The possibility of establishing this close contact with families allowed teachers to involve them directly in the student's learning, establishing formulas to transfer the support that students' needs beyond the school's hours:

We need them. But I believe, well, I am sure, that this has been something decisive and with which we have struggled the most in the 8 kindergartens (whom I supervise). The fact that a parent comes (to the school) with a specific goal, which is not to bring breakfast, nor to clean the bathroom, nor fix the desk… to let them see and have a commitment to their children's learning. Or we have many family members who are the grandfather, the uncle, the tutor... but who are clear in what they are going to give support on. And (we have) very pleasant experiences in which they have realized how to help them (Diana, Technical-pedagogical advisor, Special Education).

Nora illustrated the possibilities that emerge from this collaboration with families through the experience of Leo, a 6-year-old student with an intellectual disability enrolled in the third year of early childhood education. In the following excerpt, she explained how Leo's Special Education teacher was able to capitalize on the participation of Leo's mother in the classroom, in order to provide her with tips to reinforce her son's learning at home:

For example, in the case of Leo, (...) just something that allowed us to see the use of the different materials, and see what caught his attention and, later, the specialist teacher designed materials that she gave to her mother, to work at home. So, it's like saying: “we already work on this in Interactive Groups, he still has a little difficulty, but look, here is the material that you are going to take this week, to work with him at home, and in a week, we'll come and see if there was further progress.” So, we would meet the following week with the material and the child (...) and see if she had favored the use of the material with him. And then, she herself would say: “well, what are you going to give me now? What have you been working on with in Interactive Groups?” (Nora, Pedagogical Advisor, Special Education).

Engaging families in their children with Special Educational needs' learning not only allows them to support them more effectively outside of the classroom, but also turn family members into firsthand spectators of their children's progress, while increasing their appreciation of the work done by teachers. This was revealed in Marcelo's case when his mother began to participate regularly in Interactive Groups as a volunteer:

Marcelo's mother comes when we do Interactive Groups (...) when we finished and we asked them as volunteers what their reaction had been, what they liked and so on, she started to cry, and she said to the teacher Miguel: “Thank you very much, because I had never seen my son sitting for more than 5 min in an activity, thank you for what you have done with my son.” So, I think that these types of situations leave a mark (Diana, Technical-pedagogical advisor, Special Education).

Building and nurturing this type of collaboration with families required a significant effort on the part of teachers when it comes to involving families in the evidence-based dialogic approach that underpins their classroom practice. At the same time, it required a transformation of expectations toward the role of families in their children's learning. The participants pointed out the relevance of their gained experience through dialogic teacher education to start promoting this dialogue with families. In Miguel's words:

I think magic happens when you feel heard. In addition, when you don't go to school just to hear complaints, that you don't know how to be a parent, that your child doesn't behave well, that you don't know how to do things… (Miguel, Special Education teacher, Kindergarten).

#### Making Dialogic Training Sustainable to Keep Improving School Practice

Transforming classrooms into interactive learning environments to promote the learning and development of all students, including those with Special Educational Needs, required an ongoing effort and commitment on the part of all educational agents, which allowed them to consolidate the transformations undertaken and to deepen the improvement of educational practice. To this end, the participating teachers highlighted the continuity of dialogic teacher education—through the participation in the dialogic pedagogical gatherings within the permanent seminar—as a key formula to make the improvements promoted in their schools sustainable. This ongoing dialogic teacher education has helped participants not to lose focus of the goal that is at the heart of all these efforts: to improve the learning opportunities of all students, especially those with special needs:

The fact of attending monthly helps you (...) to maintain this link and this part of: ”Let's remember why we are in this situation, why we are dreaming this part, what we have in common“. Well, I left after the seminars, at the end, with this desire to continue, with this continuity to think about what else to propose to finally achieve these objectives that we had. That I think it helped us to have them very clear (Anita, Special Education supervisor).

Stimulating this renewed and constant commitment to evidence-based dialogic training among teachers made it possible that the transformations promoted in the school do not depend on the political initiatives of the moment, nor on the commitment of a specific group of teachers, but rather transcended them and reached the community, thus becoming part of the vision of their schools:

We have good foundations, I think we have educated ourselves and we have created a network among ourselves, and that must sustain us. (...) It is not a burden that we say: “no, as people have already changed, here we leave it, and now let's see what they give us.” On the contrary, the commitment is still there, and even greater, because perhaps there is no longer someone who is asking you for evidence of what you are carrying, but you are doing it because you are seeing the results, and you know that this is a benefit for the community (Anita, Special Education supervisor).

Participants recognized that commitment and rigor are necessary ingredients for the dialogic teacher training to become continuous and sustainable. At the same time, the creation of networks of support and collaboration between teachers has helped participants not to lose heart and cope with difficulties collectively:

If we meet on Tuesday, come rain or shine, on Tuesday we will be there. Be very, very formal with the commitment. In that case, yes, I admire Nora because, yes, she is extremely responsible in this type of task. And the days that we stayed, those days they were there. And what is the result? Well, obviously, the community joins in, the parents, the teachers, etc (Anita, Special Education supervisor).

I do believe that the entire team is willing to follow this as far as it must go (...) We are going to carry on, and for me it is a pride to say that (so shall) despite the limitations… (Diana, Technical-pedagogical advisor, Special Education).

## Discussion

The present study explored the transformative pathway undertaken by a group of in-service teachers working in the field of Special Education in Mexico. After engaging in two different dialogic teacher education programs, participants introduced changes in their educational practice with the aim of increasing the opportunities for learning and social participation of their students with Special Educational Needs enrolled in general classrooms. In addition, the study identified the forms through which the participating teachers managed to embrace this evidence-based knowledge and translate it into their daily educational practice, in order to create more inclusive and interactive learning environments for all their students, including their students with Special Educational Needs. Hence, the emphasis of dialogic teaching education on preparing participating teachers to implement interactive, evidence-based interactive learning environments had an impact on participants, helping them to redefine their practice as Special Education teachers working in mainstream schools.

While there is growing consensus regarding the relevance of creating evidence-based interactive learning environments to move toward the goal of ensuring an inclusive education for all (Pinto et al., 2019; Duque et al., 2020), segregation—usually in the form of withdrawing pupils with Special Education needs from the general classroom for support—is still a common practice in the field of Special Education in many countries (Rose and Shevlin, 2020; Somma, 2020). Faced with this reality, our study pointed to dialogic teacher education as a powerful strategy for the professional development of Special Education teachers, a field that is facing important changes (Rock et al., 2016) linked to the need to promote more transformative teacher education models aligned with the principles of inclusive education.

Our findings revealed the importance of creating spaces for dialogue and exchange that allow teachers to get familiar with evidence-based scientific knowledge, while they reflect on the role that Special Education teachers should play, to contribute to the goal of increasing the opportunities for learning and social interaction of students with Special Educational Needs within regular classrooms. Our results are in line with those of other studies placing teacher education as a critical tool to move toward more inclusive educational approaches (Robinson and Goodey, 2017; Florian and Camedda, 2020), and stressing the need to advance in the study of the tools and programs that offer better support and preparation for teachers when developing their teaching work in more inclusive contexts.

Furthermore, the study has made it possible to identify keys to transferring evidence-based knowledge regarding the relevance of interactions for learning to everyday practice in the field of Special Education, an aspect in which a gap had been identified (Cook and Odom, 2013). In this regard, the study revealed a series of strategies that have allowed participating teachers to translate the knowledge acquired through dialogic teacher education into their daily practice, in order to ground their educational actions in evidence-based knowledge. Firstly, our findings revealed how through the implementation of two evidence-based Successful Educational Actions that transform the classroom into an interactive learning environment (namely, Interactive Groups and Dialogic Literary Gatherings) they have managed to increase the interactions among students with and without Special Educational Needs, aimed at solving learning activities within the classroom. These findings coincide with those of previous studies, which have pointed to the power of Successful Educational Actions as tools that fosters inclusivity, through the social interaction between students with diverse needs (Duque et al., 2020; Zubiri-Esnaola et al., 2020). The transformation of the learning environment through the implementation of these Successful Actions not only had an impact on students with Special Educational Needs' opportunities for learning, but also on their peers, since it allowed students without special needs to actively get involved in the academic process of their peers with diverse needs, thus increasing the opportunities to maximize interactions among students in terms of quantity, quality and diversity (Aguilera-Jiménez and Prados-Gallardo, 2020). In addition, our findings revealed how, through the involvement of families and other members of the community in the classroom, teachers were able to strengthen the impact of the Successful Actions aimed at improving the learning and social outcomes of students with Special Educational needs, thus extending the impact of these interactive learning environments beyond the classroom. In line with the findings of other studies that highlight the need for teachers to join forces with key stakeholders to improve the educational response to students with special needs (Ní Bhroin and King, 2020), our study showed how engaging families in the transformation of the classroom's learning environment has been a critical tool to move from discourse to action when increasing the learning opportunities of students with Special Educational Needs. Furthermore, participants highlighted the importance of a sustainable commitment to dialogic training, in order to continue improving their educational practice through the implementation of evidence-based knowledge aimed at favoring the inclusion of their students with Special Educational Needs.

In the context of growing global agreement on the need to move toward inclusive education for all (United Nations, 2015), our study has contributed to shed light on two dialogic teacher training initiatives (initial and ongoing dialogic professional development programs) that allowed a group of teachers working in the field of Special Education to improve their preparedness to respond to the needs of Special Education students enrolled in general classrooms. Through an evidence-based dialogic approach aimed at equipping teachers with theoretical and practical tools to strengthen their collaborative work (Robinson, 2017) with general teachers and with families and other members of the community, dialogic teacher education provided an opportunity for the participants to rethink and give a new impetus to their role as teachers in the field of Special Education. In this sense, the participants' renewed vision of the centrality of Special Education teachers when it comes to transforming general classrooms into more inclusive spaces for all students—which emerged and flourished from the participation in evidence-based training—is aligned with prior research emphasizing the need to support teacher's self-efficacy when serving students with Special Educational Needs (Sharma et al., 2012; Malinen et al., 2013; Chao et al., 2017). Furthermore, our findings are in line with those of Ruppar et al. (2018), which highlighted how the efforts of teachers to value the capacities of students with diverse needs and raise expectations toward their learning possibilities have an impact on the professionalization and recognition of teachers working in Special Education. The ways in which the participating teachers detected their (prior) low expectations regarding the learning possibilities of their students with special needs and, as a consequence, started promoting transformations in the classroom (such as the implementation of evidence-based actions like Interactive Groups or Dialogic Gatherings, the participation of families in the classroom and the creation of spaces for continuous dialogic teacher education, etc.) offered an example of the impact of involving Special Education teachers in high-quality training opportunities on the improvement of their educational work with Special Education students in the general classroom.

The study has some limitations that must be noted. First, the information collected in the study is largely based on the perspectives of the participating teachers collected through in-depth interviews. In order to gain a deeper understanding of the impact of the evidence-based dialogic teacher education on the academic experiences of their students with Special Educational Needs, further studies must delve into these processes, collecting the voices of other relevant stakeholders (families, students with Special Educational needs, peers without Special Educational needs, etc.). This may allow us to provide a more nuanced and in-depth picture of the role of the different actors involved in transforming the classroom into a more inclusive learning environment. Likewise, the study focused on the results obtained after the first year of implementation of the Successful Educational Actions. Although the information analyzed provided detailed evidence of the improvements achieved in the classrooms involved in the study, more research is needed in order to analyze the evolution of these improvements over time. In addition, further research may deepen on the necessary conditions for the dialogic training of teachers to have a direct impact on daily practice in the classrooms, which may inform future evidence-based dialogic teacher training programs in the different countries which are currently implementing Successful Educational Actions. Notwithstanding its exploratory nature, the study suggests the promising impact of dialogic teacher education on the transformation of the educational practice of a group of Special Education teachers in Mexico, a country that in recent decades is making strides to establish more inclusive educational policies (Garcia-Cedillo et al., 2014; Garcia-Cedillo, 2018) and to improve teacher professionalization (Hrusa et al., 2020). The efforts of participants to align their practice with the evidence-based knowledge gained through dialogic teacher education to transform their classrooms into more interactive learning environments that embrace students' diversity illustrate the need for further research on how to improve teacher education and professional development to contribute to the shared goal of ensuring more inclusive learning environments for all.

## Data Availability Statement

The raw data supporting the conclusions of this article will be made available by the authors, without undue reservation.

## Ethics Statement

The studies involving human participants were reviewed and approved by Ethics Board of the Community of Researchers on Excellence for All (CREA). The patients/participants provided their written informed consent to participate in this study.

## Author Contributions

We declare that all authors have made substantial contributions. LR-E and AR-O contributed to the conceptualization of the study under the research line successful educational actions and schools as learning communities in the framework of the Ramon y Cajal grant (awarded to LR-E) and the AR-O's Ph.D. AR-O collected the data. PA drafted the manuscript. All authors contributed to the formal analysis, discussion of the data, and made edits for important intellectual content. LR-E and MR-S revised the final version of the manuscript. All authors approved the final manuscript.

## Conflict of Interest

The authors declare that the research was conducted in the absence of any commercial or financial relationships that could be construed as a potential conflict of interest. The reviewer L-CM declared a shared affiliation, with no collaboration, with some of the authors AR-O, MR-S, and LR-E, to the handling editor at time of review.
